# Case Report: Polyvinylpyrrolidone deposition disease from repeated injection of opioid substitution drugs: report of a case with a fatal outcome

**DOI:** 10.12688/f1000research.51927.1

**Published:** 2021-04-19

**Authors:** Ida Viken Stalund, Gro Nygard Riise, Friedemann Leh, Tormod Karlsen Bjånes, Lars Riise, Einar Svarstad, Sabine Leh

**Affiliations:** 1 Department of Pathology, Haukeland University Hospital, Post box 1, 5021 Bergen, Norway; 2 Department of Clinical Medicine, University of Bergen, Jonas Lies vei 87, 5021 Bergen, Norway; 3 Department of Medicine, Haukeland University Hospital, Post box 1, 5021 Bergen, Norway; 4 Department of Medical Biochemistry and Pharmacology, Haukeland University Hospital, Post box 1, 5021 Bergen, Norway; 5 Kvam Municipality, Grovagjelet 16, 5600 Norheimsund, Norway

**Keywords:** Polyvinylpyrrolidone, PVP, povidone, opioid substitution therapy, opioid substitution drugs, methadone, adverse effect, case report

## Abstract

**Background**: Intravenous injection of oral opioid substitution drugs (OSD) is widespread among injecting drug users. Several OSDs contain the polymer polyvinylpyrrolidone (PVP) as an excipient. Parenterally administered PVP of high molecular weight may accumulate in tissues and organs. This phenomenon was first described in the 1950s, when PVP was utilised in medication for parenteral use. We report a case of an opioid-addicted patient with extensive PVP–deposition caused by repeated injections of OSDs.

**Case presentation**: A 30-year-old male drug addicted patient in opioid substitution therapy (OST) was repeatedly referred to his local hospital in a poor general condition. Work-up revealed severe normocytic anaemia, renal insufficiency, pancreas insufficiency and pathological fractures. Biopsies from fractured bones, bone marrow and gastric mucosa showed extensive infiltrates of histiocytes with intracytoplasmic vacuoles. Vacuole content stained slightly bluish in hematoxylin and eosin stain, red in Congo red stain and black in periodic acid methenamine silver stain. The morphological appearance and staining properties were in accordance with the diagnosis of PVP deposition. The patient had been injecting both buprenorphine tablets and a specific methadone syrup for several years. The methadone syrup contained large amounts of high molecular weight PVP, making it the most likely cause of the deposition. His health quickly deteriorated and he died, impaired by multi-organ failure and cachexia, five years after the first diagnosis of PVP-deposition. The autopsy revealed extensive PVP-deposition in all sampled organs and tissues.

**Conclusions**: Histological investigation and the correct identification of PVP in the biopsies led to the discovery of a severe adverse effect from long-standing misuse of a drug. The disseminated PVP deposition likely contributed to multi-organ dysfunction and cachexia with a fatal outcome. The deposited PVP likely originated from repeated injections of a certain methadone syrup.

## Background

Injection of oral opioid substitution drugs (OSD) is a concern in the treatment of opioid dependency. An Australian study found that 7–13% of clients in opioid substitution therapy (OST) injected their medication weekly or more often. ^1^ The OSDs may also be sold on the illegal drug market, and 26.6% of out-of-treatment intravenous drug users in a Norwegian study reported having injected methadone during the past 4 weeks. ^2^ Injection of oral or sublingual drug formulations may lead to vascular and soft tissue damage with a range of secondary complications. ^3^

Several OSDs contain the excipient polyvinylpyrrolidone (PVP), ^4^ a water-soluble polymer with a wide variety of applications in the pharmaceutical industry. ^5^ When orally ingested, PVP is not absorbed, and causes no harm. ^6, 7^ When injected, PVP is not metabolized, and the only way of excretion is via glomerular filtration. ^6^ While low molecular weight (MW) PVP is freely filtered by the glomerulus, PVP with moderate or high MW will be partly or completely retained in the body. ^6, 8^ In the middle part of the last century, PVP was utilized as a plasma expander ^9^ and as a retarding agent in hormone preparations for injection. ^10^ Reports from this time described storage in multiple tissues following repeated parenteral administrations of PVP-containing preparations. ^9– 11^

We report a case of extensive PVP-deposition disease with a fatal outcome following long-term injection of PVP-containing OSDs.

## Case presentation

### First admission

A male, 30-year-old drug addicted patient in OST was admitted to the local hospital. He was hepatitis C positive and had a history of hospitalisations for skin infection. At admittance, he was in a poor general condition with nausea, vomiting, abdominal pain and muscle aches. Physical examination revealed a diffusely tender abdomen and poor dental status. Laboratory investigations disclosed non-specific inflammatory signs with an increased erythrocyte sedimentation rate (83 mm/h) and C-reactive protein (CRP, 90 mg/L), severe normocytic anaemia with a haemoglobin of 7.8 g/dL and renal insufficiency (serum creatinine 133 μmol/L, estimated glomerular filtration rate 60.1 mL/min/1.73m ^2^) with microalbuminuria. Blood cultures were negative. Radiological examinations of the thorax and abdomen showed splenomegaly and a pancreatic cyst, but otherwise no radiologic signs of infection, malignancy or kidney pathology. His CRP and serum creatinine levels fell spontaneously, and he left the hospital against the doctor’s advice after four days.

### Second admission

**Figure 1.**
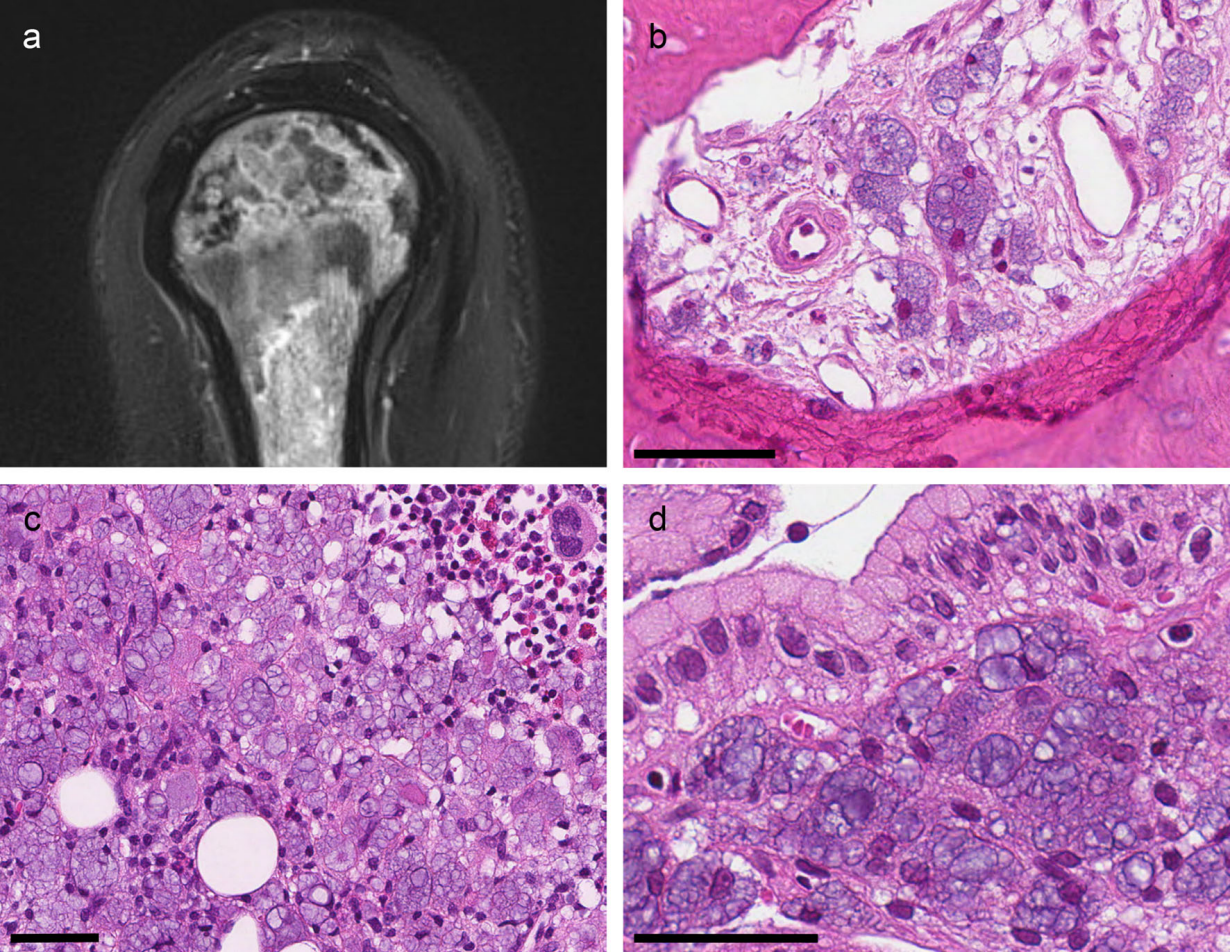
Radiological and biopsy findings. (a) MRI (T2-blade-sag-FS) of the right humerus showing mottled signal changes. (b) Clavicular bone (H&E): The marrow space is infiltrated by histiocytes with bluish transparent bubbles. (c) Bone marrow (H&E): Massive infiltration of histiocytes and scarce remaining hematopoietic tissue. (d) Gastric mucosa (H&E): Infiltration of vacuolated histiocytes in an extended lamina propria. All scale-bars 50 μm.

Two months later, the patient fractured his right clavicle. After a two-week delay, he was admitted to hospital with a fever and a swollen and erythematous clavicular region. His general condition and nutritional status had worsened, nausea and vomiting persisted and anaemia and renal insufficiency had relapsed. Blood cultures were positive for Staphylococcus aureus, and antibiotic treatment against suspected osteomyelitis was initiated. Magnetic resonance imaging (MRI) of the clavicular region and the upper arm showed mottled signal changes with a high signal intensity in the lateral clavicle, the humeral bone and the acromion ( Figure 1a). The diagnoses considered at this time were osteomyelitis or malignancy. Biopsies from the fractured bone, bone marrow and gastric mucosa were performed in the work-up of this complex symptomatology.

### Biopsy findings

The biopsies all showed similar infiltrates of histiocytes with a cytoplasm extended by vacuoles of different sizes ( Figure 1b– d) and eccentrically located nuclei.

Biopsies from the fractured bone revealed reactive changes with ongoing fibrosis. The fibrotic tissue contained the multivacuolated histiocytes as singular cells, small groups or sheets of cells ( Figure 1b). The bone marrow biopsy showed massive histiocytic infiltrates ( Figure 1c). There was reduced fat cell content, and there was almost no visible hematopoietic tissue. The gastric biopsy showed antrum mucosa with elongated gastric pits and aggregates of multivacuolated histiocytes in both the superficial and deep lamina propria ( Figure 1d). A gastric biopsy taken two years previously was re-examined. It showed the same histiocytic infiltrates; however, the findings did not lead to the correct diagnosis at the time.

**Figure 2.**
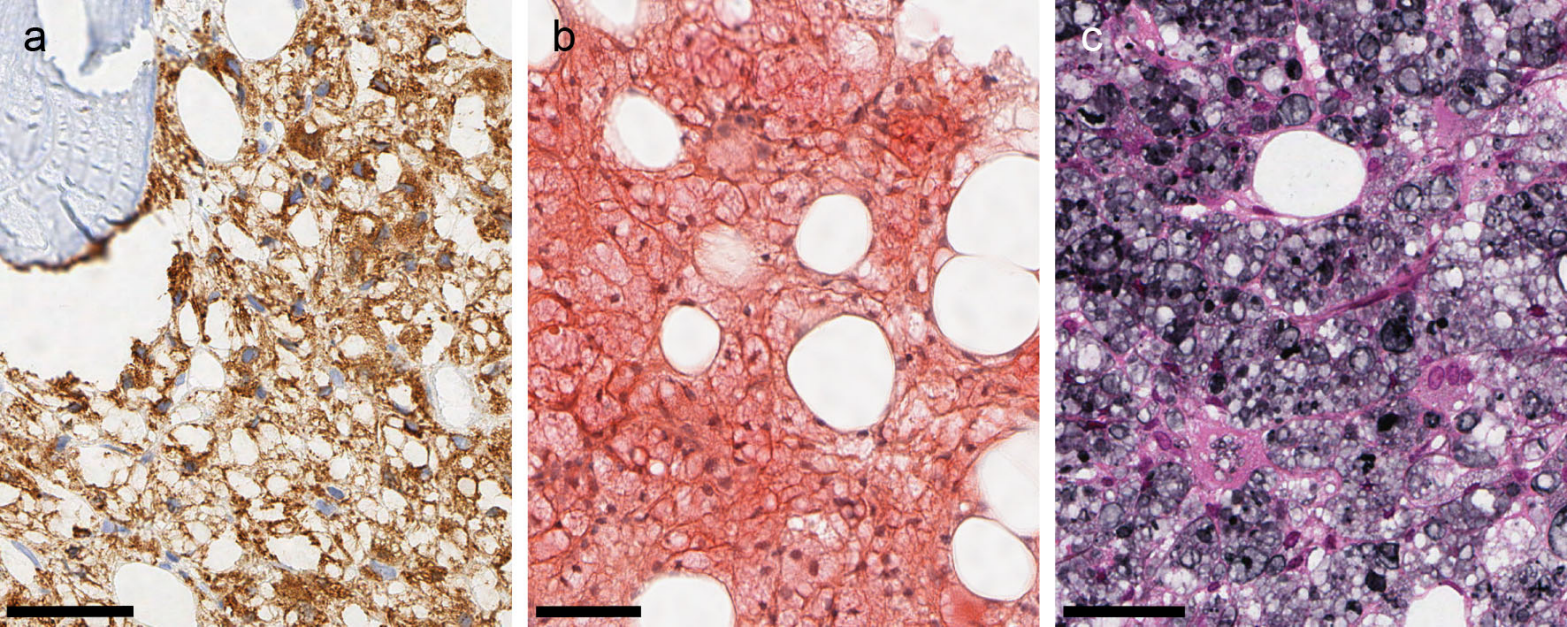
Staining properties. All micrographs are from the same bone marrow biopsy. (a) CD 68/PGM1: The vacuolated cells are CD68-positive histiocytes. (b): Congo red stain: Vacuole content stains faintly red. (b) PASM: Vacuole content stains grey or black. All scale bars 50 μm.

In the hematoxylin and eosin (H *&*E) stain, the vacuoles had distinct membranes and a bluish, transparent looking content. Vacuole content did not stain in the periodic acid Schiff (PAS), Prussian blue or Alcian blue stain. The cells were positive for CD68/PGM1 confirming the histiocytic nature ( Figure 2a). The vacuoles stained red in Congo red stain and black in the periodic acid methenamine silver (PASM) stain ( Figure 2b and c). The vacuoles did not show birefringence.

### Interpretation

The microscopic appearance of the vacuolated histiocytes and the histochemical staining properties were consistent with PVP deposition. ^12, 13^ At that time, several of the OSDs marketed in Norway contained PVP. ^4^ Our patient had injected both buprenorphine tablets and methadone syrup regularly over several years. The buprenorphine tablets contained PVP K30 (MW 44–54 kDa). The specific methadone syrup he had been injecting contained large amounts of PVP K90 (MW 1 000–1 500 kDa). ^14^ While much of the PVP K30 is expected to be excreted within weeks, PVP K90 will not be excreted and consequently accumulates in the body. ^6^ It is therefore plausible that injections of the PVP K90-containing methadone syrup were the cause of the PVP-deposition disease in this patient. Based on the history of this patient and other similar cases, ^15, 16^ the European Medicines Agency suspended this methadone syrup in 2014. ^14^

### Further development

After the diagnosis was made, the patient’s health gradually declined, in part due to his poor self-care and underlying chronic drug addiction. There is no specific treatment for PVP deposition disease. His persistent anaemia was treated with regular blood transfusions and erythropoietin-stimulating agents with limited effect. The kidney failure was managed by supportive treatment, and serum creatinine levels fluctuated between 150 and 450 μmol/L.

**Figure 3.**
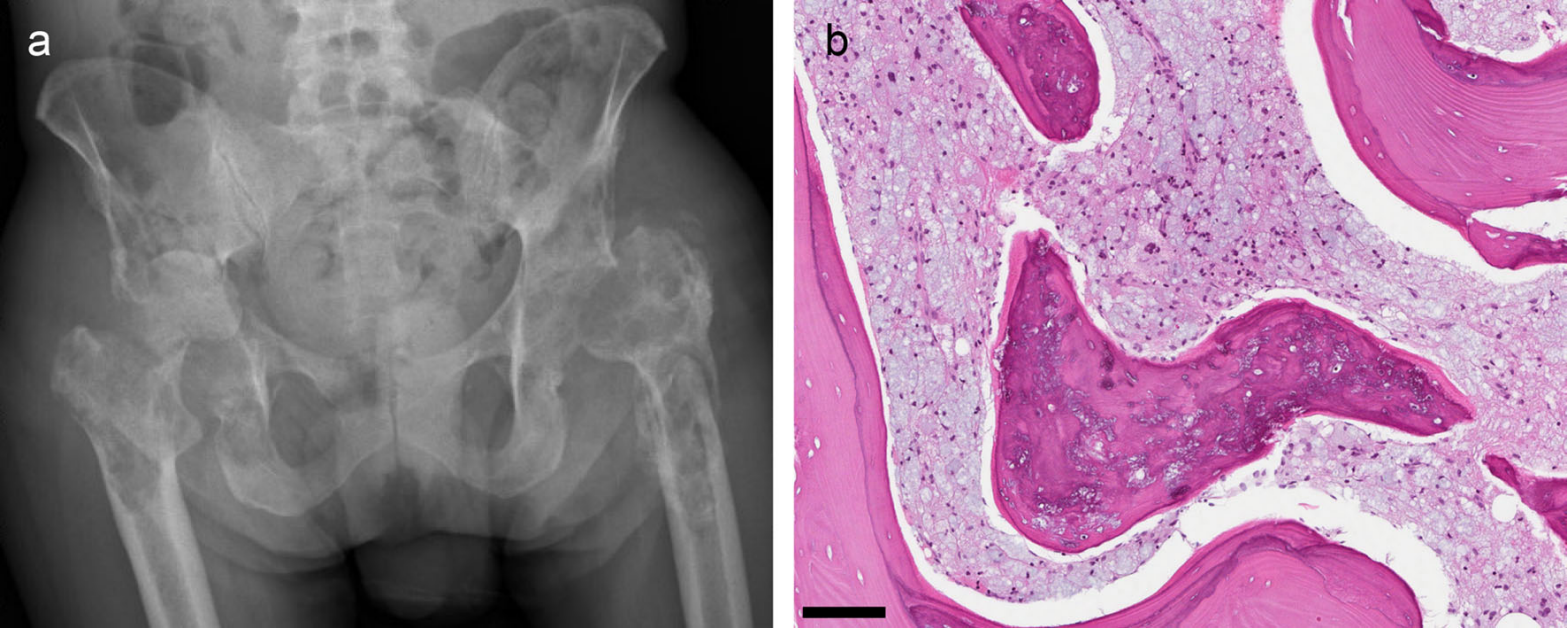
Pathological fractures and bone destruction: radiological and biopsy findings. (a) Pelvic radiograph. Left hip: The hip prosthesis has been removed due to loosening and replaced by a Girdlestone hip. Right hip: Extensive lytic and sclerotic changes in the proximal femur and acetabulum leading to medial dislocation of the femoral head. (b) Biopsy from the right greater trochanter (H&E): The marrow space is filled with histiocytes with the bluish vacuoles characteristic of PVP-deposition. The bone trabecula has empty lacunar spaces and contains a bluish material. Scale bar 50 μm.

Within the first year of the diagnosis, the patient suffered a left sided pathological femoral neck fracture with impaired fracture healing, complicated by chronic osteomyelitis. In the right hip, he developed severe bone destruction of the acetabulum with dislocation of the femoral head and a subsequent femoral neck fracture ( Figure 3a). MRI scans of both hips and the pelvis (not shown) revealed the same changes as those detected in the shoulder region. Biopsies from the greater trochanter region showed extensive histiocytic infiltrates ( Figure 3b). Surgical treatments were unsuccessful. Henceforth his mobility deteriorated to such a degree that he needed a wheelchair and required daily activity support and care in a palliative care centre. Furthermore, he developed endocrine pancreas insufficiency with insulin-dependent diabetes mellitus. He had a gradual weight loss of 22 kg during four years despite adequate food intake. Malabsorption due to exocrine pancreas insufficiency was suspected, but supplementation of pancreas enzymes had little effect on the weight loss.

Five years after the diagnosis of PVP deposition disease, the patient died, impaired by multi-organ failure and advanced cachexia with a body mass index below 15 kg/m ^2^.

### Autopsy

**Figure 4.**
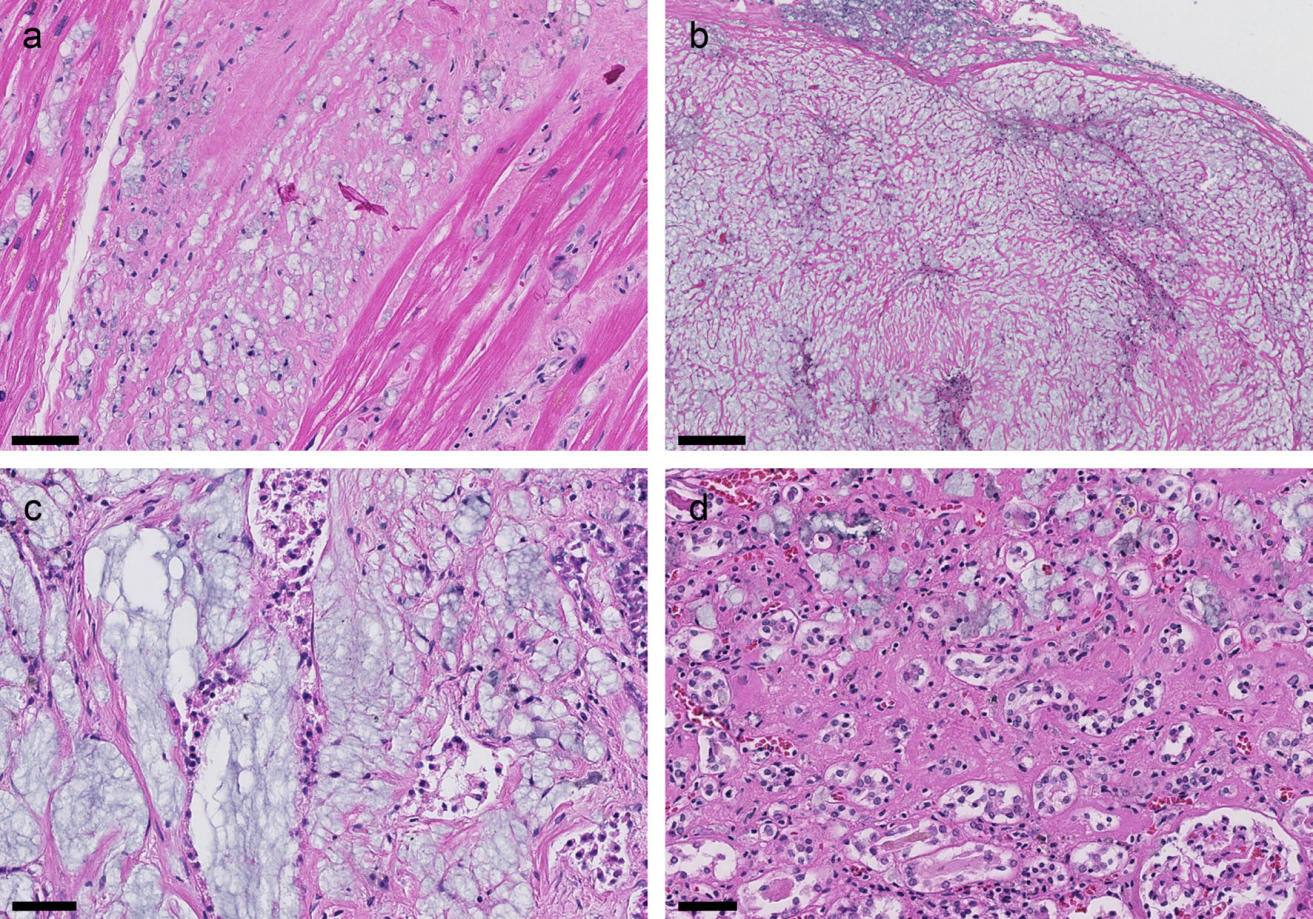
Autopsy findings (H&E). Infiltrates of histiocytes with the bluish vacuoles characteristic of PVP in all organs. (a) Myocardium: Interstitial fibrosis and histiocytic infiltrates. Scale bar 50 μm. (b) Pleura: Nodular lesion composed of fibrotic tissue and histiocytes. We found similar lesions in the pericardium and peritoneum. These were macroscopically visible. Scale bar 200 μm. (c) Pancreas: Pronounced fibrosis with histiocytic infiltrates. Poorly preserved pancreatic parenchyma. Scale bar 50 μm. (d) Kidney: Cortical tissue showing interstitial fibrosis, tubular atrophy, and an infiltrate of histiocytes in the extended interstitium. Scale bar 50 μm.

The autopsy concluded that the immediate cause of death was multi organ failure due to PVP deposition disease, which in turn was a consequence of the patient’s illicit substance abuse. The following organs and tissues were sampled during the autopsy: the pericardium and myocardium, the pleura and lungs, kidneys, liver, pancreas, gastric mucosa, adrenal glands, peritoneum and abdominal adipose tissue, the spleen, lymph nodes, and femoral bone and bone marrow. Microscopically, we observed PVP-deposition in all sampled organs and tissues ( Figure 4). The PVP-containing histiocytes were partly scattered in the interstitium, e.g. in the myocardium ( Figure 4a), and partly organised like larger, nodular lesions, e.g. in the pleura ( Figure 4b). Peri- and intra-neural distribution was seen in several organs. Autopsy findings corresponded well with the clinically observed pancreas and kidney insufficiency. In the pancreas, dense fibrosis was interspersed by heavy infiltrations of histiocytes ( Figure 4c). There was little preserved pancreas parenchyma. The kidney showed moderate to severe interstitial fibrosis and tubular atrophy, and only minor glomerular changes. There were infiltrates of PVP-containing histiocytes in the interstitium, mostly in atrophic areas ( Figure 4d). There were no amyloid depositions or signs of other renal diseases.

## Discussion

We present the case of a drug-addicted patient with widespread PVP-deposition disease. We describe the patient’s clinical course from the first diagnosis of PVP-deposition to his death five years later and present biopsy and autopsy findings. The PVP deposition disease in this patient was likely caused by repeated intravenous injections of a methadone syrup containing high MW PVP.

The characteristic appearance and staining properties of PVP in tissue samples are well known. ^17^ Positivity for Congo red and PASM and negativity for PAS distinguishes PVP deposits from those seen in hereditary storage diseases. The histiocytic nature of the cells and the PAS negativity rule out metastatic signet ring cell carcinoma as a differential diagnosis. ^17^ Furthermore, PVP is not birefringent, unlike most other foreign materials commonly observed in tissue samples from injecting drug users. ^18^ Hence, making the diagnosis of PVP deposition is straightforward if this option is considered.

Whether PVP deposition causes disease was controversial for a long time. The first reports of PVP- deposition described storage in the tissue that persisted years after the administration of PVP, but disputed whether the storage was harmful to the functioning of the target organs. ^19^ Later reports described clinically relevant adverse effects. Those most frequently reported were cytopenias, bone destruction, polyneuropathy and granulomatous lesions of the skin. ^17, 20– 23^ PVP deposition in internal organs such as the liver, kidneys, pancreas and the gastrointestinal tract has also been described, but the adverse effect of the deposition in these organs is less well known. ^24, 25^

Our patient experienced a complex clinical syndrome and rapidly deteriorating health. The reason for his health decline was multifactorial, and his continued drug addiction likely aggravated the clinical course. Many of the clinical conditions associated with the fatal outcome correspond to findings in biopsies and the autopsy showing extensive PVP deposition. We believe that the extensive PVP deposition contributed to his health decline through several mechanisms. The widespread PVP deposition in bone and bone marrow was likely the main reason for the patient’s anaemia, pathological fractures and impaired fracture healing, fitting well with previous reports. ^20, 22, 23^ The impaired mobility and chronic osteomyelitis that resulted from these fractures gravely affected his health and quality of life. Furthermore, progressive cachexia was an important part of his health decline. The reason for the weight loss was not established during his lifetime, but his continued drug use likely contributed. Other possible contributing factors were pancreas insufficiency, progressive kidney failure, malabsorption, chronic infections and continued problems with vomiting, all likely related to the extensive PVP deposition. In summary, the PVP deposition probably played a major role in causing the patients’ multiple organ dysfunction ultimately leading to the fatal outcome.

Injection of oral OSDs is common among injecting drug users and has long been a concern in the treatment of opioid addiction. ^2^ As an attempt to prevent injections, the previously mentioned methadone syrup was made highly viscous. ^14^ However, the increased viscosity did not prevent such unintended use. As a consequence, the choice of high MW PVP as thickener caused further severe adverse effects from injection.

## Conclusions

This case revealed an unanticipated explanation for anaemia and pathological fractures in a drug-addicted patient. The correct identification of the observed foreign material as PVP revealed that injection of a certain methadone syrup containing PVP probably caused the patient’s deposition disease. Based on the clinical history, biopsy and autopsy findings, we conclude that the widespread PVP deposition likely contributed to the patient’s severe morbidity and death.

## List of abbreviations

**PVP:** Polyvinylpyrrolidone

**OSD:** Opioid substitution drug

**OST:** Opioid substitution therapy

**H&E:** Hematoxylin and eosin stain

**PASM:** Periodic acid methenamine silver stain

**PAS:** Periodic acid Schiff stain

**CRP:** C-reactive protein

**MRI:** Magnetic resonance imaging

**MW:** Molecular weight

## Data availability

All data underlying the results are available as part of the article and no additional source data are required.

## Reporting guidelines

Zenodo: CARE checklist for “Polyvinylpyrrolidone deposition disease from repeated injection of opioid substitution drugs: report of a case with a fatal outcome”. https://doi.org/10.5281/zenodo.4667989. ^26^

Data are available under the terms of the Creative Commons Zero “No rights reserved” data waiver (CC0 1.0 Universal).

## Consent for publication

Written informed consent for publication of the patient’s clinical details and clinical images was obtained from the patient prior to his death and from the closest relative.
