# Supplementary material for: Case Report: Polyvinylpyrrolidone deposition disease from repeated injection of opioid substitution drugs: report of a case with a fatal outcome
Source: F1000Res. 2021 Jul 7;10:300. Originally published 2021 Apr 19. [Version 2] doi: 10.12688/f1000research.51927.2 (PMC8276182; doi:10.12688/f1000research.51927.2)
Supplement: Peer review history

# Peer review: Reviewer response for version 2

**DOI:** 10.5256/f1000research.58229.r89200

**Published:** 2021-07-08

**Authors:** Maike Büttner-Herold

**Affiliations:** 1 Department of Nephropathology, Institute of Pathology, Friedrich-Alexander University Erlangen-Nuremberg (FAU), Erlangen, Germany

The authors addressed all points I had to my perfect satisfaction and I have no further questions. Thank you for the opportunity to review this very interesting case.

Are enough details provided of any physical examination and diagnostic tests, treatment given and outcomes?

Partly

Is the case presented with sufficient detail to be useful for other practitioners?

Yes

Is sufficient discussion included of the importance of the findings and their relevance to future understanding of disease processes, diagnosis or treatment?

Yes

Is the background of the case’s history and progression described in sufficient detail?

Yes

Reviewer Expertise:

Nephropathology, GvHD, immunology

I confirm that I have read this submission and believe that I have an appropriate level of expertise to confirm that it is of an acceptable scientific standard.

# Peer review: Reviewer response for version 1

**DOI:** 10.5256/f1000research.55139.r85062

**Published:** 2021-05-26

**Authors:** Robert Barnes Colvin

**Affiliations:** 1 Department of Pathology, Massachusetts General Hospital, Boston, MA, USA

The paper shows no new information, but the problem is a major one, with missed diagnoses and serious consequences. I think the paper will receive some attention with the major opioid crisis we have.

 I have no specific suggestions, other than a careful editing of the prose and the addition of at least on other major publication which has embolized material (EM) - e.g., Ganesan *et al.* (2003) ^1^.

Are enough details provided of any physical examination and diagnostic tests, treatment given and outcomes?

Yes

Is the case presented with sufficient detail to be useful for other practitioners?

Yes

Is sufficient discussion included of the importance of the findings and their relevance to future understanding of disease processes, diagnosis or treatment?

Yes

Is the background of the case’s history and progression described in sufficient detail?

Yes

Reviewer Expertise:

NA

I confirm that I have read this submission and believe that I have an appropriate level of expertise to confirm that it is of an acceptable scientific standard.

## References

[1] : Embolized crospovidone (poly[N-vinyl-2-pyrrolidone]) in the lungs of intravenous drug users. *Mod Pathol* .2003;16(4) : 10.1097/01.MP.0000062653.65441.DA 286-92 10.1097/01.MP.0000062653.65441.DA 12692192

# Response

**Published:** 2021-06-28

**Authors:** Ida Viken Stalund

**Affiliations:** Haukeland University Hospital, Norway

We thank you for your thorough review of our manuscript.

 To address your comment, we have added the following sentence in the second paragraph of the discussion: “PVPs light blue color in H&E and the typical localization of the deposits differentiates it from the non-water soluble variant of PVP, crospovidone.” and added your suggested reference.

# Peer review: Reviewer response for version 1

**DOI:** 10.5256/f1000research.55139.r83519

**Published:** 2021-04-28

**Authors:** Maike Büttner-Herold

**Affiliations:** 1 Department of Nephropathology, Institute of Pathology, Friedrich-Alexander University Erlangen-Nuremberg (FAU), Erlangen, Germany

The present case report illustrates a very impressive case of PVP deposition in histiocytes in a large number of organs in a patient who repeatedly injected oral opioid substitution drugs. The description of such a finding is very valuable as the identification of foreign material can be very difficult for a pathologist, if the histological picture has not previously been seen. The illustration of the findings is very nicely done.

 Some minor points could be addressed:

- Besides the reported anaemia did the patient also have thrombopenia and leukopenia, which could explain the infectious complications?
- Was the patient free of infectious complications when he died?
- Can a loss of pancreatic islets be shown by immunhohistochemistry? In Figure 4C one wonders whether remaining islets are depicted.
- As PVP is filtered in the glomeruli, were histiocytes detecable in the glomeruli? Was the distribution of PVP-loaden histiocytes in the kidneys dependent on the renal compartment?
- Did the cardiac infiltration by histiocytes lead to functional impairment of the cardiac output or to arrhythmias?
- Is their a possible explanation why PVP should interfer with the structure of the bone in such a destructive way?

- Does the lack of birefringence of PVP also apply to the Congo red staining?
- Keywords: It would maybe be helpful to include "foreign material" and "histiocytic storage" to facilitate search for a pathologist finding PVP in a patient without being able to assign the finding to the right cause.

Are enough details provided of any physical examination and diagnostic tests, treatment given and outcomes?

Partly

Is the case presented with sufficient detail to be useful for other practitioners?

Yes

Is sufficient discussion included of the importance of the findings and their relevance to future understanding of disease processes, diagnosis or treatment?

Yes

Is the background of the case’s history and progression described in sufficient detail?

Yes

Reviewer Expertise:

Nephropathology, GvHD, immunology

I confirm that I have read this submission and believe that I have an appropriate level of expertise to confirm that it is of an acceptable scientific standard, however I have significant reservations, as outlined above.

# Response

**Published:** 2021-06-28

**Authors:** Ida Viken Stalund

**Affiliations:** Haukeland University Hospital, Norway

We thank you for your thorough review of our manuscript. We have addressed your comments point by point.

*Besides the reported anemia, did the patient also have thrombocytopenia and leukopenia, which could explain the infectious complications?*

- The patient had episodes of mild thrombocytopenia and leukopenia. In general, his leukocyte response to infection was weak. We added the following sentence in the section “Further development” to address this question: “He had short episodes of mild thrombocytopenia and leukocytopenia, and in general, his leukocyte response to infection was weak.”

*Was the patient free of infectious complications when he died?*

- In the days and weeks prior to his death, he had stable, moderately increased CRP. Clinically, he showed no signs of acute infection and he did not receive antibiotic treatment. The autopsy revealed small foci of acute inflammation in the pancreas, but otherwise there were no signs of an acute infection. We added the following sentence in the section “Autopsy” to address this question: "The autopsy revealed no evidence of infection besides minor foci of acute inflammation in the pancreas."

*Can a loss of pancreatic islets be shown by immunohistochemistry? In Figure 4C, one wonders whether remaining islets are depicted.*

- Based on H&E morphology there was little preserved pancreatic parenchyma, both pancreatic acini and islets. Synaptophysin and Chromogranin A staining revealed a marked loss of pancreatic islets. CK-19 staining revealed areas of some preserved ductal structures, though the morphology was impaired by autolysis.
  We changed the following sentence in the section “Autopsy” to address this question: "There was little preserved exocrine and endocrine pancreas parenchyma. The pancreatic tissue was dominated by dense fibrosis interspersed by heavy infiltrations of histiocytes (Figure 4c)."
- Figure 4C depicts fibrosis, PVP-containing histiocytes and ductal structures impaired by autolysis. Synaptophysin and Chromogranin A staining could not detect islets in this area. We changed the legend to figure 4C accordingly: (c) Pancreas: Pronounced fibrosis with histiocytic infiltrates. Poorly preserved ductal structures.

*As PVP is filtered in the glomeruli, were histiocytes detecable in the glomeruli? Was the distribution of PVP-loaden histiocytes in the kidneys dependent on the renal compartment?*

- In H&E, we observed occasional PVP-containing vacuoles in glomeruli. With CD68 staining, we detected histiocytes in most glomeruli. The number of histiocytes per glomerulus cross section was variable, but always less than ten. We added the following sentence in the section “Autopsy” to address this question: In glomeruli, we observed a slightly increased number of histiocytes and occasional PVP-containing vacuoles. 
- The vast majority of PVP-containing histiocytes were seen in the tubulointerstitial compartment, as stated in the “Autopsy” section. 

*Did the cardiac infiltration by histiocytes lead to functional impairment of the cardiac output or to arrhythmias?*

- The autopsy revealed infiltrates of PVP-containing histiocytes in the myocardium. We also observed peri- and intraneural distribution of the histiocytes. It is possible that such infiltrates could induce arrhythmias or other forms of cardiac impairments. Our patient did not show signs of such organ dysfunction, neither clinically nor in echocardiographic and electrocardiographic evaluations. We added the following sentence in the section “Autopsy” to address this question: “Peri- and intra-neural distribution was seen in several organs, including the heart. However, there were never any clinical, echocardiographic or electrocardiographic evidence of impaired cardiac output or arrhythmias prior to his death.” 

*Is there a possible explanation why PVP should interfere with the structure of the bone in such a destructive way?*

- The only mentioning of a possible cause for the bone destruction is in in a case report from 1993: Kepes *et al.* suggested that cells containing engulfed PVP would undergo mucoid alterations as a result of interactions between the engulfed material and the cytoplasm. The authors argue that in bone, the PVP-induced mucoid changes cause “softening of the bone” and interfere with the structural integrity of the bone trabeculae. One could also speculate that the massive infiltration of histiocytes in the marrow space could affect the blood supply to the osseous tissue, causing a form of avascular necrosis.
  We think a discussion of this unresolved matter is out of focus for this case report.

*Does the lack of birefringence of PVP also apply to the Congo red staining?*

- Yes, the lack of birefringence of PVP also apply to the Congo red. We changed the following sentence in the section “Biopsy findings” to address this question: “The vacuoles did not show birefringence in Congo red stain or any other stains.”

*Keywords: It would maybe be helpful to include "foreign material" and "histiocytic storage" to facilitate search for a pathologist finding PVP in a patient without being able to assign the finding to the right cause*

- Good point. We added these keywords.
